# Effective Suppression of Methane Emission by 2-Bromoethanesulfonate during Rice Cultivation

**DOI:** 10.1371/journal.pone.0142569

**Published:** 2015-11-12

**Authors:** Tatoba R. Waghmode, Md. Mozammel Haque, Sang Yoon Kim, Pil Joo Kim

**Affiliations:** 1 Division of Applied Life Sciences (BK 21 PLUS program), Gyeongsang National University, Jinju 660–701, South Korea; 2 Netherlands Institute of Ecology (NIOO-KNAW), Department of Microbial Ecology, Wageningen, The Netherlands; 3 Institute of Agriculture and Life Sciences, Gyeongsang National University, Jinju 660–701, South Korea; Chinese Academy of Sciences, CHINA

## Abstract

2-bromoethanesulfonate (BES) is a structural analogue of coenzyme M (Co-M) and potent inhibitor of methanogenesis. Several studies confirmed, BES can inhibit CH_4_ prodcution in rice soil, but the suppressing effectiveness of BES application on CH_4_ emission under rice cultivation has not been studied. In this pot experiment, different levels of BES (0, 20, 40 and 80 mg kg^-1^) were applied to study its effect on CH_4_ emission and plant growth during rice cultivation. Application of BES effectively suppressed CH_4_ emission when compared with control soil during rice cultivation. The CH_4_ emission rates were significantly (*P*<0.001) decreased by BES application possibly due to significant (*P*<0.001) reduction of methnaogenic biomarkers like Co-M concentration and *mcrA* gene copy number (i.e. methanogenic abunadance). BES significantly (*P*<0.001) reduced methanogen activity, while it did not affect soil dehydrogenase activity during rice cultivation. A rice plant growth and yield parameters were not affected by BES application. The maximum CH_4_ reduction (49% reduction over control) was found at 80 mg kg^-1^ BES application during rice cultivation. It is, therefore, concluded that BES could be a suitable soil amendment for reducing CH_4_ emission without affecting rice plant growth and productivity during rice cultivation.

## Introduction

Methane (CH_4_) is the second most important greenhouse gas after carbon dioxide (CO_2_) and its annual contribution to global warming is about 40% [1]. Large amount of CH_4_ is released to the atmosphere as the end product of archaeal metabolism under anaerobic condition [2]. The major anaerobic sites of CH_4_ production are rice paddies, ruminants, natural wetlands and sediments [3]. Rice paddies contribute ca. 5–19% of the total global CH_4_ emissions and may increase further due to the expansion of rice cultivation to fulfill the demand of an increasing human population [1].

Rice (*Oryza sativa* L.) is the major food crop for people living in Asia, and about 80% of rice grown are under submerged conditions [4]. Flooding of rice field promotes anaerobic degradation of plants derived carbon by methanogens, which results in CH_4_ production [5]. All methanogens synthesize coenzyme M (Co-M) as an methyl group carrier during CH_4_ biosynthesis process, where methyl group carried by Co-M is reduced to CH_4_ by methyl-Co-M reductase (MCR) enzyme [6]. 2-bromoethanesulfonate (BES) is a structural analogue of Co-M and a potent inhibitor of methanogenesis [7], which makes competitive inhibition with Co-M for methyl group and subsequently can inhibit methanogens CH_4_ production activity (i.e. MCR enzyme activity). Therefore, CH_4_ production can be effectively suppressed by controlling the concentrations of Co-M and *mcrA* gene copy number (gene encoding for alpha subunit of MCR enzyme) abundance in soil. The inhibition of methanogenesis by BES under anaerobic condition has been well established, however, to date available study on effect of BES on CH_4_ dynamics (mainly, CH_4_ production potential) in paddy soils is without rice plant [8–10]. The hypothesis of this study was that BES application might be effective to mitigate CH_4_ emission from rice soil; however, its effect on soil chemical properties and rice plant growth was not known as it was the first attempt to use BES in rice paddy soil for mitigating CH_4_ emission. In this experiment, three different doses of BES were applied in rice paddy soil under greenhouse condition and changes in CH_4_ emission fluxes were correlated to the soil chemical and biochemical properties. The objective of this study was to evaluate the possibility of using BES for mitigating CH_4_ emission from rice paddy soils.

## Material and Methods

### Experimental set-up

The pot experiment was conducted in a greenhouse at agricultural farm of Gyeongsang National University, Jinju, South Korea. Soil was collected from rice field (0–15 cm depth) in the spring of 2013. The soil sample was air dried, sieved (<10 mm) and packed into Wagnor pot (25 cm in diameter and 30 cm in height, 13 kg dried soil pot^-1^). The soil collected for this experiment was fine silty, mixed, mesic Typic Endoaquept [11]. The soil had following characteristics: organic matter, 10.88±1.61 g kg^-1^; total N, 0.74±0.42 g kg^-1^; soil pH, 6.68±0.26 (soil: H_2_O = 1:5, w/v), available phosphate, 45.06±0.49 mg kg^-1^ and exchangeable cations Ca^2+^, Mg^2+^ and K^+^, 3.58±0.33, 0.60±0.04 and 0.35±0.03 cmol^+^ kg^-1^, respectively. Pots were then flooded with water and allowed to stand for stabilization (filling up of capillary pores with water). After 1 week of flooding, chemical fertilizer and 2-bromoethanesulfonate (BES) were applied and 25 days old 3 seedlings of Korean rice cultivar ‘Dongjinbyeo’ (*Oryza sativa*, Japonica type) was transplanted (June 20, 2013) in each pot.

The chemical fertilizers were applied at the rates of 90 kg N ha^-1^, 45 kg P_2_O_5_ ha^-1^, and 58 kg K_2_O ha^-1^ as per the Korean recommended fertilization levels for rice cultivation [12], using urea, fused superphosphate and potassium chloride. The basal chemical fertilizer applied before transplanting were: 45 kg N ha^-1^, 45 kg P_2_O_5_ ha^-1^ and 40.6 kg K_2_O ha^-1^. Tillering fertilizer (18 kg N ha^-1^) was broadcasted approximately 2 weeks after rice transplanting and panicle fertilizer (27 kg N ha^-1^, 17.4 kg K_2_O ha^-1^) was broadcasted 6 weeks after rice transplanting. BES was applied at different levels as 0 (control), 20, 40 and 80 mg kg^-1^ of soil. The BES concentration selected in this experiementis on the basis of incubation test results, where 80 mg kg^-1^ BES (soil weight basis) showed ca. 50% inhibition of CH_4_ production in rice soil (data not shown). The ‘bases’ of cylindrical chambers were permanently fixed in each pot and then the pots were arranged in the greenhouse following completely randomized design. Each treatment had three replicates. The water level was maintained at 5–6 cm above the soil surface during cropping season and then drained 2 weeks before rice harvesting. The harvesting of rice was carried out after 120 days after transplanting (hereafter, DAT).

### Gas sampling

A closed-chamber method was used to measure CH_4_ emissions from rice planted pots during rice cultivation [13]. The gas collection chambers having a diameter of 24 cm and height of 100 cm with a circulating fan for gas mixing and thermometers to monitor inside temperature were placed on bases of rice planted pots during gas sampling. The air gas samples were collected from chambers using 50 ml air-tight syringes at 0, 15 and 30 min intervals after chamber placement and transferred into pre-evacuated 20 ml glass vials fitted with butyl rubber stoppers for analysis in the laboratory. Gas sampling was carried out once a week and three times (0800, 1200 and 1600 h) in day to get the average CH_4_ emission flux during cropping season. Gas sampling and air temperature measurements were simultaneously carried out.

### Measurement of CH_4_ concentrations

CH_4_ concentrations in the collected air samples were measured by gas chromatography (Shimadzu, GC-2010, Japan) packed with a Porapak NQ column (Q 80–100 mesh) and a flame ionization detector (FID). The temperatures of column, injector and detector were adjusted at 70°C, 150°C and 200°C, respectively. Helium and hydrogen were used as carrier and burning gases, respectively. Average fluxes and standard deviations were calculated from triplicate pots.

Methane emission from soil was calculated as the increase in CH_4_ concentrations per unit surface area of the chamber for a specific time interval. A closed-chamber equation was used to estimate CH_4_ fluxes from each treatment [13].

$$\text{F}=p\times \frac{V}{A}\times \frac{\Delta c}{\Delta t}\times \frac{273}{T}$$

Where, F was the CH_4_ flux (mg CH_4_ m^-2^ hr^-1^), *ρ* was the gas density (0.714 mg cm^-3^), V was the volume of the chamber (m^3^), A was the surface area of the chamber (m^2^), Δc/Δt was the rate of CH_4_ gas accumulation in the chamber (mg m^-3^ hr^-1^), and T (absolute temperature) was calculated as 273 + mean temperature in (°C) of the chamber.

Total CH_4_ flux for the entire cultivation period was calculated using following equation [14].

$$Total\;CH4\;flux\;\left(g\;m-2\;d-1\right)=\sum_{i}^{n}\left(\text{Ri}\times \text{Di}\right)$$

Where, R_i_ was the CH_4_ emission flux (g m^-2^ d^-1^) in the *i* ^th^ sampling interval, D_i_ was the number of days in the *i* ^th^ sampling interval, and n was the number of sampling intervals.

### Coenzyme M concentration in soil

To determine Co-M concentration, the fresh soil collected on 30 DAT (active tillering), 60 DAT (Booting), 80 DAT (Heading) and 120 DAT (Harvesting) was homogenized with lysis buffer (100 mM Tris-HCl solution (pH 8.0), 100 mM EDTA solution (pH 8.0) and 1.5 M NaCl solution) (Soil: buffer = 1: 2, w/v basis) and sonicated for 2 min (1 min sonication followed by 10 sec vortex and then 1 min sonication again). The soil suspension was centrifuged at 4000 rpm for 10 min. The required amount of absolute ethanol was added to the 2 ml supernatant to make it 80% ethanol solution. The solution mixture was allowed to stand for 2 h at 4°C and centrifuged again at 4000 rpm for 10 min. The precipitate was dissolved in deionized water and diluted to a suitable volume for high performance liquid chromatography (HPLC) analysis. 10 μl of the serially diluted standard solutions were injected into the column (Agilent Eclipse XDB—C_18_, 4.6 x 250 mm) of HPLC (Agilent DE/1200, 5 μm) and the data were analyzed at 270 nm wavelength using UV detector. The mixture of acetonitrile and 50 mM trichloroacetic acid solution (30:70, v/v) was used as mobile phase for Co-M quantification.

### Extraction of soil DNA and PCR amplification

The soil samples collected at 30, 60, 80 and 120 DAT during rice cultivation were immediately lyophilized by Pilot Lyophilizer (PVTFD50A, Ilsin, Korea) and then sieved through 2-mm size. The DNA was extracted from the lyophilized soil samples by using FastDNA SPIN Kit for Soil (MP Biomedical, CA, USA) following the manufacturer’s instructions. The extracted DNA was used as a template for PCR to amplify *mcrA* gene (alpha subunit of methyl coenzyme M reductase) using suitable primers [15], mlas_forward (5’-GGTGGTGTMGGDTTCACMCARTA-3’) and *mcrA*_reverse (5-CGTTCATBGCGTAGTTVGGRTAGT-3’). The PCR amplification was performed with a Takara Extaq (Takara biotechnology, Japan) using 1 μl of a DNA template in 25 μl of reaction mixture. The PCR amplification was performed with the following reaction conditions: initial denaturation at 95°C for 3 min, 34 cycles of 95°C for 45 sec, annealing at 55°C for 45 sec and 72°C for 45 sec, followed by a final extension at 72°C for 7 min. The PCR product was analyzed by electrophoresis on a 1.2% agarose gels to verify the extraction and amplification. DNA concentrations were quantified by Nanodrop 2000 spectrophtometer (Thermo Scientific, USA).

### Quantitative PCR targeting *mcrA* genes

The quantitative PCR of *mcrA* gene copy numbers were analyzed by BioRad CFX96 real-time thermocycler (BioRad Laboratories, Hercules, CA, USA). The reaction mixture (SYBR Green Real-time PCR Master Mix, Toyobo, Japan) was composed of 10 pmol of each primer [15], 1 μl template DNA (10 ng μl^-1^) and sterilized distilled water added to make the final volume up to 40 μl. The initial denaturation was done at 95°C for 3 min, followed by 40 cycles at 95°C for 45 sec, 55°C for 45 sec and 72°C for 45 sec. The DNA standard was prepared from the purified plasmid DNA of *mcrA* clone after 10-fold serial dilutions of plasmids containing a sequence of *mcrA* gene from *Methanosarcina mazei*. The amplification efficiency of the PCR was calculated using standard curves with the following formula:

Efficiency=[10(−1/slope)]−1

The amplifications of serial diluted standards were performed for samples of each pot to minimize the inhibitory effect exerted by substances co-extracted with DNA. The quality of the amplification was evaluated by the generation of a melting curve for the PCR product.

### Methanogens and soil dehydrogenease activity

In order to determine the effect of BES on methanogenesis, methanogens activity was carried following method of Pramanik and Kim [16]. The soil samples were collected at 30, 60, 80 and 120 DAT in each treatment pot in triplicate during rice cultivation. Ten gram of fresh soil was mixed with 25 ml distilled water in 115 ml serum bottle and incubated under anaerobic condition at 30±0.5°C for 5 h. The methanogen activity was measured by estimating CH_4_ concentration in the headspace of the bottles and the values were expressed as ng of CH_4_-C produced g soil^-1^ hr^-1^.

In order to check the effect of BES application on soil enzyme activity (i.e. soil biological activity) other than methanogenesis, the soil dehydrogenase activity was monitored during rice cultivation. The soil dehydrogenase activity was determined using the reduction of 2,3,5-triphenyltetrazolium chloride (TTC) method [17]. A sample of 6 g soils and 60 mg CaCO_3_ were mixed thoroughly and then were transferred into each of three glass vails (20 ml). To each vial with stopper, 1 ml of 3% TTC and 2.5 ml of deionized water were added. The samples were mixed on a vortex and incubated at 37°C. After 24 h, the triphenylformazan (TPF), a product from the reduction of TTC, was extracted by adding 10 ml methanol and shaken for 1 min. The samples were collected in a volumetric flask. The vial was washed with methanol until the red color disappeared. The filtrate was then diluted with additional methanol to a final volume of 100 ml. The color intensity was measured at 485 nm with methanol as a blank.

### Investigation of soil properties, rice plant growth and yield characteristics

Soils were collected in triplicate (from each treatment) from the 0–15 cm depth at the harvesting stage, air dried, and passed through a 2-mm size sieve for chemical analysis. The soil chemical properties were analyzed using the Korean standard method [18]: pH (1:5 with H_2_O), available phosphate (Lancaster method), organic matter content (Walkley and Black method; [19]) and total N [18]. At harvesting stage, the whole rice plant was cut from the 1–2 cm above soil surface from each pot and transferred to the lab for yield parameter measurement. A rice plant growth parameters like plant height, tiller numbers, straw yield and rice yield characteristics like ripened grains %, 1000 grain weight and grain yield and total biomass were investigated at a harvesting stage of rice plant. Yield components were determined by following Korean standard rice cultivation guidelines [20].

### Statistical analyses

Statistical analyses were conducted using SPSS 11.5 software for windows. A one-way analysis of variance (ANOVA) was carried out to compare the means of the different treatments. Tukey’s post-hoc test was used to separate treatment means when the F-test showed to be significant at the *P*<0.05 probability level. Linear regression analysis was performed to evaluate relationships between response variables.

## Results

### CH_4_ emissions from rice paddy soils

**Fig 1:**
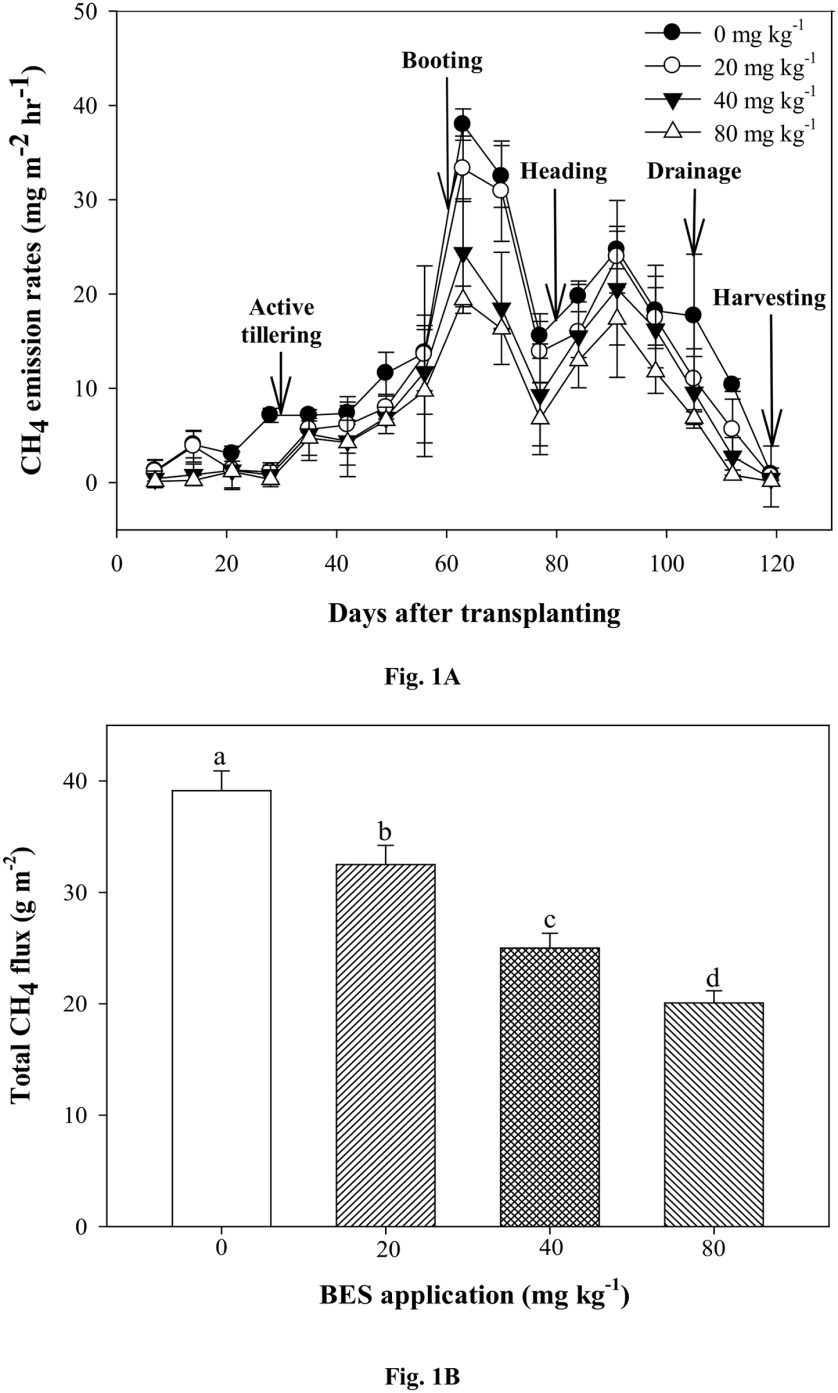
Changes in CH_4_ emission rates with time (A) and total CH_4_ fluxes (B) under different levels of BES application during rice cultivation. Error bar indicates standard deviation (n = 3; mean ± SD). Different letters indicate significant difference according to Tukey’s post-hoc test (*P*<0.05).

CH_4_ emissions were gradually increased after transplanting and showed first peak at 63 DAT followed by second peak at 91 DAT, thereafter decreased prior to the rice harvest (Fig 1A). The highest CH_4_ emission was recorded in control treatment and BES application effectively (*P*<0.001) reduced rate of CH_4_ emission during rice cultivation. The rate of CH_4_ emissions were inversely proportional to the doses of BES application. The total seasonal CH_4_ flux from rice planted soils were significantly affected by BES applicaiton. The total CH_4_ flux in control soil was 39.2 g m^-2^, which was significantly decreased by 17–49% after BES application (Fig 1B).

### Coenzyme M concentration in soil

**Fig 2:**
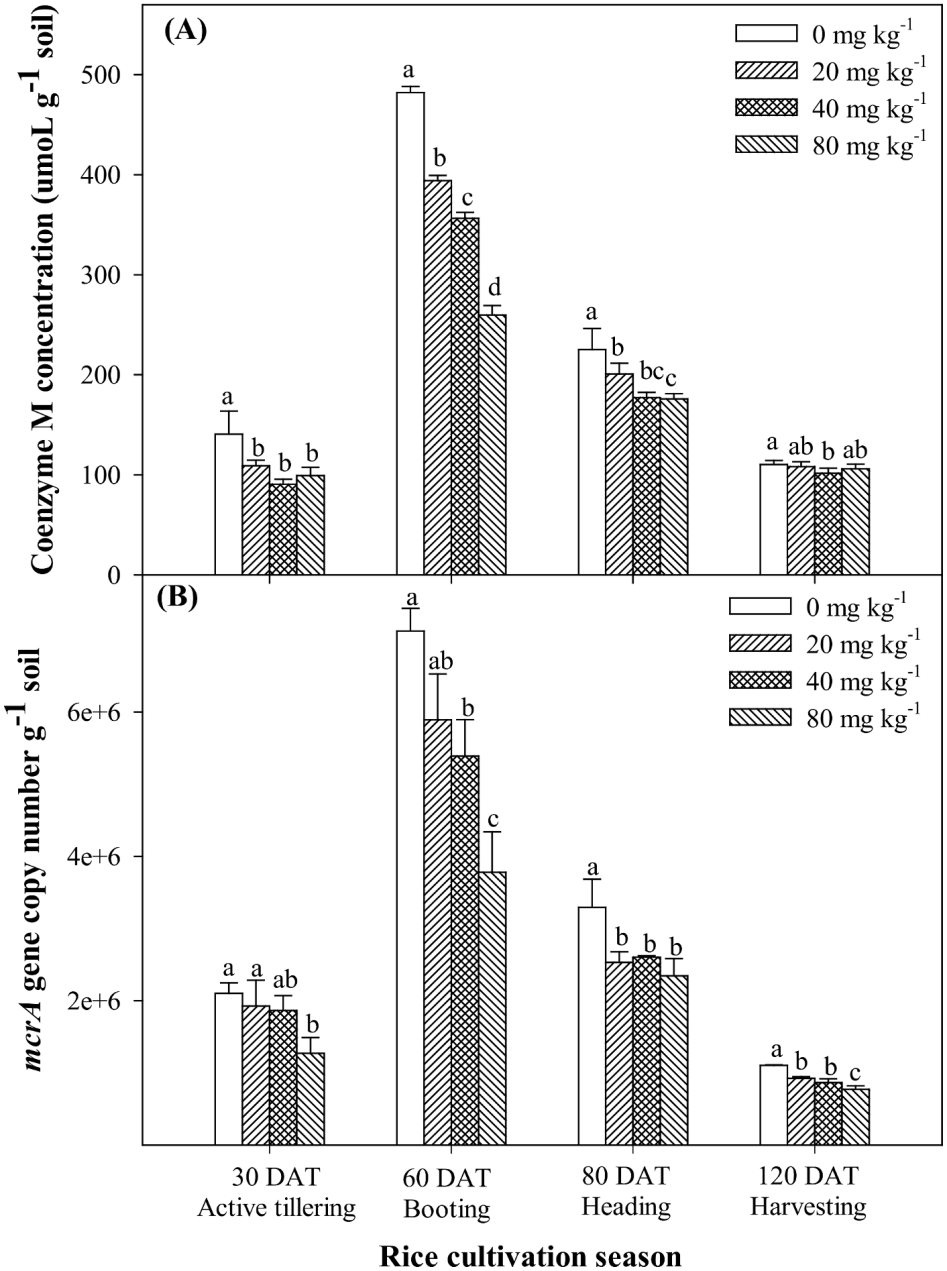
Changes of coenzyme M concentrations (A) and *mcrA* gene copy number (B) in rice paddy soils under different levels of BES application during rice cultivation. Error bar indicates standard deviation (n = 3; mean ± SD). Different letters indicate significant difference according to the Tukey’s post-hoc test (*p*<0.05).

Coenzyme M concentrations in soil varied depending on the applied treatments and rice cultivation period. Irrespective of the time of rice cultivation, the highest Co-M concentrations were observed in control soil and BES application significantly (*P*<0.001) reduced Co-M concentration in soil (Fig 2A). At active tillering stage, Co-M concentrations in 20 and 40 mg mg kg^-1^ treament soils were statistically at par with 80 mg kg^-1^ BES treatments, which was significantly (*P*<0.05) increased at booting stage. At booting stage, Co-M concentration in the control soil was 482.1 ± 6.07 μmoL g soil^-1^, which was decreased to 394.1 ± 5.15, 356.4 ± 5.94 and 259.7 ± 9.4 μmol g soil^-1^ in 20, 40 and 80 mg kg^-1^ BES treatment soils, respectively.

### Methanogenic abundance in soil

Irrespective of rice cultivation, the highest *mcrA* genes abundance was observed in the control and the application of BES significantly (*P*<0.001) decreased *mcrA* genes in soil (Fig 2B). The *mcrA* genes of 20 and 40 mg kg^-1^ BES treatment soils were higher than that of 80 mg kg^-1^ treated soil, but lower than the control soil during rice cultivation. At booting stage, the *mcrA* gene copy numbers of 80 mg kg^-1^ treated BES soil were significantly lower (*P*<0.05) when compared to the control soil.

### Methanogens and soil dehydrogenase activity

Methanogen activity in rice paddy soils also varied with the BES application levels and period of rice cultivation (Table 1). BES application significantly (*P*<0.001) reduced methanogen activity in soil and recorded lower methanogen activity at all rice cultivation stages. At booting stage, methanogen activity was highest in control soil and the BES application significantly (*P*<0.05) reduced in it.

**Table 1:** Enzyme activities in soil at varying cultivation stages of rice plant tested in paddy fields with different levels of BES application.

Enzyme activities	BES application (mg kg^-1^)	Rice cultivation stages			
		Active tillering (30 DAT)	Booting (60 DAT)	Heading (80 DAT)	Harvesting (120 DAT)
^a^ **Methanogen activity**	0	21.4 ± 6.34a	126.2 ± 7.2a	55.0 ± 4.15a	25.0 ± 15.3a
	20	19.7 ± 5.19a	101.1 ± 6.84b	48.0 ± 2.35ab	22.1 ± 7.97a
	40	17.6 ± 1.99a	93.2 ± 12.2b	38.0 ± 5.61b	20.4 ± 1.98a
	80	12.8 ± 0.66a	61.0 ± 5.04c	35.0 ± 6.21b	15.5 ± 0.42a
^b^ **Dehydrogenase activity**	0	6.51 ± 1.29a	14.7 ± 1.10a	8.07 ± 0.52a	3.33 ± 0.30a
	20	6.65 ± 0.62a	15.3 ± 0.95a	8.20 ± 0.88a	3.52 ± 0.29a
	40	6.94 ± 1.13a	15.1 ± 1.02a	8.16 ± 0.54a	3.72 ± 0.14a
	80	7.02 ± 1.39a	14.8 ± 2.43a	8.13 ± 0.67a	3.62 ± 0.26a

Note: Values in the same column followed by same letters are not significantly different at p<0.05, ANOVA with Tukey’s post-hoc test for separation of means. Means ± SD from three replicates for each determination.

^a^Enzyme unit is ng of CH_4_-C g^-1^ soil hr^-1^

^b^Enzyme unit is μg of TPF g^-1^ soil hr^-1^

In case of soil dehydrogenase activity, the dehydrogenase activity was not affected by BES application (Table 1). The dehydrogenase activity were significantly (*P*<0.05) lower at active tillering stage than the booting stage among all treatments, but statistically at par among treatments at all stages of rice cultivation.

### Investigation of soil chemical properties, plant growth and yield characteristics

Soil chemical properties were not affected by BES application in paddy soil (Table 2). Also, BES application did not affect plant and yield characteristics, except for plant hight and straw yield.

**Table 2:** CH_4_ flux per grain yield, soil and rice plant growth and yield characteristics with different levels of BES at harvest.

Parameters	BES application (mg kg^-1^)			
	0	20	40	80
CH_4_ flux per grain yield (mg g^-1^)	75.1 ± 2.12a	60.9 ± 1.69b	47.3 ± 1.78c	40.1 ± 1.36d
**Soil properties**				
pH (1:5 with H_2_O)	6.83 ± 0.01a	6.84 ± 0.13a	6.94 ± 0.07a	6.94 ± 0.06a
Organic matter (g kg^-1^)	10.3 ± 0.58a	10.5 ± 0.63a	10.7 ± 0.59a	10.1 ± 0.47a
Total N (g kg^-1^)	0.63 ± 0.07a	0.63 ± 0.07a	0.58 ± 0.12a	0.58 ± 0.01a
Available P_2_O_5_ (mg kg^-1^)	34.9 ± 3.67a	34.9 ± 0.94a	34.6 ± 2.36a	33.1 ± 1.11a
**Plant growth and yield characteristics**				
Plant height (cm)	90 ± 0.71b	94 ± 1.0a	94 ± 1.0a	95 ± 0.58a
Tiller number per hill	20.6 ± 2.31a	20.6 ± 0.71a	21 ± 2.83a	21 ± 1.41a
Ripened grain (%)	81.5 ± 1.37a	82.7 ± 1.14a	82.4 ± 0.83a	81.6 ± 0.58b
Weight of 1000 grains (g)	19.4 ± 0.37a	19.6 ± 0.28a	19.3 ± 0.35a	19.2 ± 0.17a
Number of grains per panicle	84.2 ± 21.5a	91.1 ± 22.1a	88.1 ± 7.14a	86.6 ± 4.02a
Grain yield (g pot^-1^)	26.1 ± 0.74a	26.7 ± 0.75a	26.5 ± 1.05a	25.0 ± 0.91a
Straw yield (g pot^-1^)	55.3 ± 0.57b	55.3 ± 0.98b	56.7 ± 0.76b	58.7 ± 0.76a
Total biomass (g pot^-1^)	81.4 ± 0.91a	82 ± 0.86a	83.2 ± 0.76a	83.7 ± 1.30a

Note: Values in the same row followed by same letters are not significantly different at p<0.05, ANOVA with Tukey’s post-hoc test for separation of means. Means ± SD from three replicates for each determination.

## Discussion

Rice is generally cultivated under submerged field condition, especially in Asian countries. Continuous flooding shifted soil redox to the reduced condition, which favors the methanogenesis in soil. Vogels et al. [6] reported Co-M as a terminal CH_3_ group carrier during CH_4_ biosynthesis, and therefore, limited availability of Co-M to methanogens due to BES application could affect CH_4_ emission from soil. This means limited bioavailability of Co-M could suppress the activity of MCR enzyme in methanogens and which in turn can reduce the rate of methanogenesis in soil. However, the effect of BES application on methanogenesis in rice paddy soil was not known.

The pot experiment suggested that BES application effectively (*P*<0.001) suppressed CH_4_ emission (49% reduction in total CH_4_ flux when compared with control soil) (Fig 1A), without affecting plant growth and soil chemical and biochemical properties. The highest CH_4_ emission was found between 60–91 DAT in all treatment, but BES treatment soils showed lower CH_4_ emission than that of control soil. The highest CH_4_ emission at reproductive stage which could be due to the increased availability of substrate by root exudation for the activity of methanogens and enhanced conductivity of CH_4_ via rice plant [21, 22].

**Fig 3:**
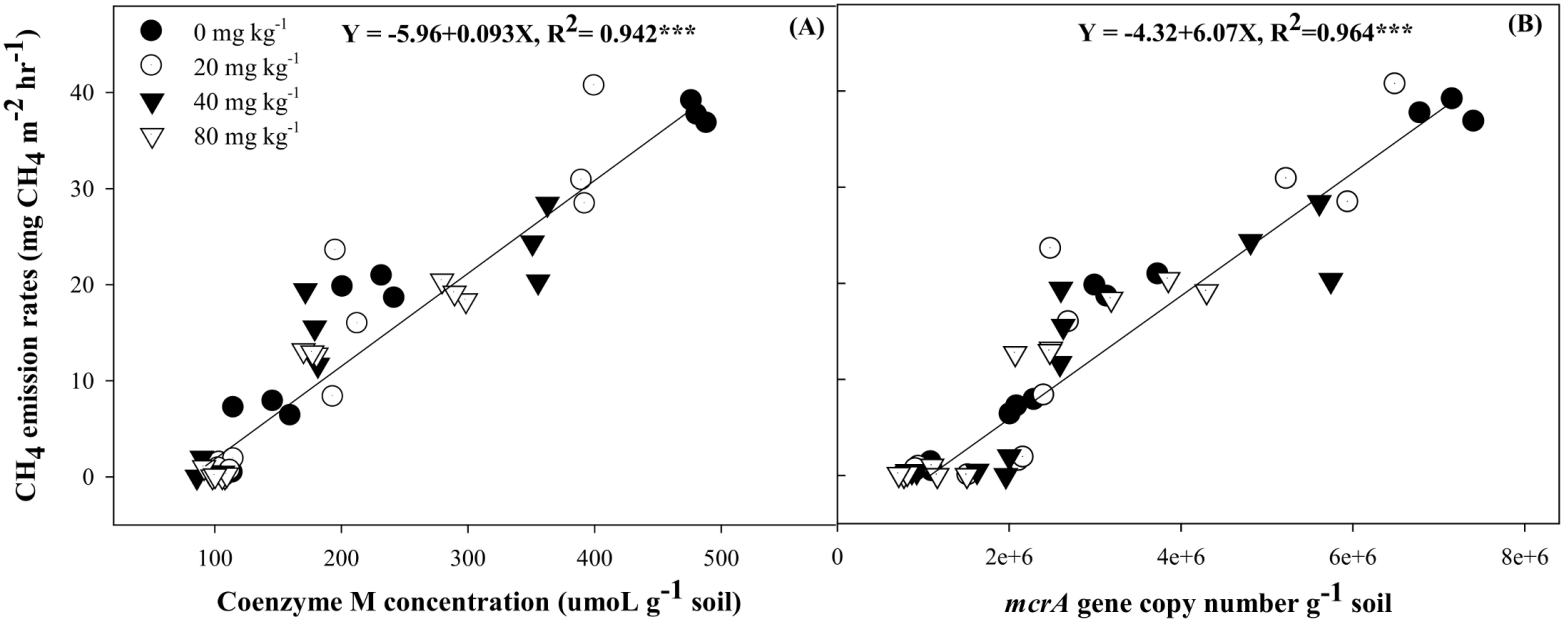
Relationships between CH_4_ emission rates, coenzyme M concentration (A) and *mcrA* gene copy number (B) during rice cultivation.

Coenzyme M and *mcrA* gene copy numbers are the biomarkers of methaogens [23] and Co-M only found in methanogens. BES is a structural analogue of Co-M and application of it makes competitive inhibition for methyl group during methanogenesis and thereby inhibits methanogenesis. Previous study reported that the application of BES inhibited methanogenesis by all species of methanogens without affecting other microbial activity in soil without rice plant [10, 24, 25]. Co-M concentration in soil could be a controlling factor for suppression of CH_4_ emission in paddy soil. Konisky [26] reported that the external application of Co-M can reverse the inhibitory effect of BES on methanogens. It means that CH_4_ emission rates could be directly proportional to the Co-M concentration in methanogens. In this study, the high positive correlation (*R* ^2^ = 0.942***) was found between Co-M concentration and CH_4_ emission during rice cultivation (Fig 3A). Application of BES at 20 and 40 mg kg^-1^ decreased Co-M concentration in soil when compared to that of control soil; however, the maximum decrease in Co-M concentration was observed in 80 mg kg^-1^ treated BES soils. Pramanik and Kim [27] also found a positive correlation between Co-M concentration and decreased CH_4_ emission with EDTA (non-specific inhibitor of methanogens) application during rice cultivation. Vogels et al. [6] reported Co-M as a methyl group carrier during CH_4_ production, and therefore, concentration of Co-M likely affect MCR enzyme activity in methanogens. The *mcrA* gene (gene coding for the alpha subunit of MCR enzyme) copy number has been used as a biomarker to detect abundance and/or activity of methanogens in paddy soil [15, 28]. In this study, the abundance of *mcrA* genes were highest in control soil and application of BES significantly (*P*<0.001) decreased *mcrA* genes during rice cultivation. The pot experiment showed CH_4_ emission rates had high positive correlations (*R* ^*2*^ = 0.964***) with *mcrA* genes during rice cultivation (Fig 3B). Likewise, Kim et al. [29] and Gutierrez et al. [30] also found a high positive correlation between *mcrA* gene copy numbers and CH_4_ emission during rice cultivation. Thus, the decrease in *mcrA* genes might be responsible for reduction in methanogens activity in soil. Similarly, Zhou et al. [31] reported that the BES addition effectively reduced total methanogen population in in-vitro ruminal cultures. Also, Morris et al. [32] found significantly positive correlation between the *mcrA* gene copy number and CH_4_ production rates. Methanogens convert simple organic C compounds into CH_4_ through enzyme mediated multi-step process [33]. BES application significantly (*P*<0.001) reduced methanogen activity without affecting other enzyme activity during rice cultivation. Likewise, previous study also found significant inhibition of methanogens activity without affecting other microbial community at 25 mM BES application [25]. Soil enzymes are considered sensitive to disturbances in paddy ecosystem. Among all enzymes in the soil environment, dehydrogenases are used as an indicator of overall soil biological activity [34], because they occur intracellular in all living microbial cells [35]. Application of BES doesn’t affect soil dehydrogenase activity during rice cultivation. It confirmed that the BES only specifically inhibits methanogenic activity without affecting other soil biological activity.

The soil properties, rice plant growth and yield characteristics were not significantly affected by BES application, except plant height and straw yield (*P*<0.05) at 80 mg kg^-1^ BES application (Table 2). Plant height, tiller numbers, straw yield and number of grains per panicles were found negative correlations with total CH_4_ flux (Table 3). In constrast, Sass [36] found a positive and significant correlation between CH_4_ and apparent growth characteristics of rice plant, because the plant’s photosynthetic carbon was used as substrate by methanoges in the rhizosphere [37]. However, our results suggest that the application of BES was responsible to reduce the methanogens activity and related CH_4_ emission. Of yield component, 1000 grain weight and grain yield were showing positive correlation with total CH_4_ flux. To estimate the combined impacts of BES addition with different levels on CH_4_ emissions and rice yield, CH_4_ flux per unit grain yield was calculated from total CH_4_ flux divided by grain yield (Table 2). This impact was significantly (*P*<0.05) decreased with the increasing BES application, mainly due to reducing total CH_4_ emission. Therefore, it could be concluded that the BES effectively reduced CH_4_ emission without affecting rice productivity in rice planted paddy soils.

**Table 3:** Correlation between total CH_4_ flux, soil properties, rice plant growth and yield characteristics.

Parameters	Correlation (r) (n = 11)
**Soil properties**	
Organic matter	0.041
Total N	0.425
Available P_2_O_5_	0.280
**Rice plant growth and yield characteristics**	
Plant height	-0.844***
Tiller number per hill	-0.132
Ripened grains %	0.424
1000 grain weight	0.633*
Number of grains per panicle	-0.041
Grain yield	0.499
Straw yield	-0.822***

Note: * and *** denote significant at 5 and 0.1% levels, respectively.

## Conclusion

The application of BES significanlty suppressed CH_4_ emission without affecting rice plant growth and crop productivity during rice cultivation. BES application at 80 mg kg^-1^ found 49% reduction in total CH_4_ flux. The decrease in CH_4_ emission by BES application could be due to decrease in concentrations of coenzyme M and abundance of *mcrA* gene copy numbers in soil. BES application significantly decreased methanogenic activity without affecting soil dehydrogenase activity. Based on these findings, application of BES effectively reduced CH_4_ emission during rice cultivation and could be used as soil amendment to supress CH_4_ emission from rice planted soils.
